# Witnessing, Embodying, and Connecting: A Phenomenological Study of Playback Theatre

**DOI:** 10.7759/cureus.94507

**Published:** 2025-10-13

**Authors:** Daisuke Son, Munenori Shiozawa

**Affiliations:** 1 Department of Community-Based Family Medicine, Faculty of Medicine, Tottori University, Yonago, JPN; 2 Department of Medical Education Studies, International Research Center for Medical Education, Graduate School of Medicine, The University of Tokyo, Tokyo, JPN; 3 Division of Home-Visit Nursing, Hibari-no-Mori Home-Visit Nursing Station Tama, Kawasaki, JPN

**Keywords:** embodiment, empathy, expressive arts therapy, phenomenological analysis, playback theatre, self-awareness, social connection

## Abstract

Background: Playback Theatre is a participatory form of improvisational theatre that allows participants to share personal stories, which are then reenacted by performers in real time. Despite its increasing use in diverse contexts, such as education, healthcare, and social work, empirical research on its psychological and social impact remains limited. This study aimed to explore participants’ lived experiences of Playback Theatre, focusing on (1) how it fosters self-awareness and personal insight, (2) how it enhances understanding and empathy toward others, and (3) its overall psychological and social impact.

Methods: This study uses Interpretative Phenomenological Analysis (IPA) to investigate how individuals experience and interpret their participation in Playback Theatre. Forty-four participants took part in six sessions held in Tokyo, representing a diverse range of backgrounds, including healthcare, education, the arts, and individuals with no theatre experience. Written reflections were collected from all participants and analyzed thematically.

Results: Three core dimensions were identified: (1) self-awareness and personal transformation, encompassing experiences of emotional release and self-discovery; (2) reflections on others and interpersonal resonance, highlighting empathy and mutual understanding; and (3) overall reflections on the Playback Theatre experience, emphasizing the role of safety, embodiment, and the uniqueness of each session.

Conclusions: This study positions Playback Theatre as a psychologically transformative and socially enriching practice. It contributes to the fields of expressive arts therapy, embodiment in emotional processing, and the psychology of storytelling, offering valuable insights for practitioners in therapeutic, educational, and community settings.

## Introduction

Playback Theatre is a distinctive form of improvisational theatre that facilitates deep personal reflection, emotional expression, and interpersonal connection. Unlike traditional theatre, Playback Theatre is interactive and participatory, allowing individuals to share personal narratives, which are then reenacted by performers in real time. This process creates a reflective and supportive space where participants not only see their experiences externalized but also engage with the lived experiences of others. As a result, Playback Theatre has gained recognition as a tool for psychosocial exploration, therapeutic intervention, and community building [1-4].

Emerging in the 1970s, Playback Theatre was founded by Jonathan Fox and Jo Salas as a theatrical form centered on personal storytelling and collective meaning-making [1]. It is rooted in the belief that storytelling is a fundamental human practice that preserves personal and collective memory while fostering connection, validation, and catharsis. The spontaneity and embodied nature of Playback Theatre set it apart from traditional performance styles, as it creates an immediate, interactive experience in which audience members become co-creators of the theatrical process [1-4].

The psychological effects of Playback Theatre can be understood through several theoretical lenses. First, expressive arts therapy and psychodrama provide a framework for understanding how enacted storytelling allows individuals to externalize and reprocess emotional experiences. Moreno’s psychodrama theory suggests that dramatization enables individuals to gain cognitive and emotional distance from their lived experiences, fostering deeper insight and psychological integration [5,6]. Similarly, research on expressive arts therapy highlights the transformative power of storytelling and improvisation as alternative forms of emotional communication, particularly for individuals who struggle with verbal expression [7].

Second, embodiment and non-verbal communication play a central role in Playback Theatre’s impact. The theory of embodied cognition posits that cognition and emotional processing are not confined to the brain but are deeply connected to bodily experiences and movements [8]. Research on embodied cognition suggests that physically enacting an experience can enhance understanding, enabling individuals to process emotions in ways that traditional verbal reflection may not facilitate [8]. This aligns with studies on movement-based approaches to trauma healing and emotional regulation, which indicate that non-verbal expression can serve as a powerful tool for emotional release and self-awareness [9].

Another relevant framework is social mirroring and empathy development. Neuroscientific research has demonstrated that observing others’ emotions activates corresponding neural pathways in the observer’s brain, fostering a sense of shared experience and empathy [10]. This phenomenon, known as mirroring, explains how witnessing another person’s story can evoke strong emotional resonance, leading to a greater understanding of one’s own experiences. Moreover, the collective aspect of Playback Theatre reinforces social attunement, as participants engage in shared storytelling that highlights commonalities in human experiences, reducing feelings of isolation and strengthening emotional connections [11].

Given its emphasis on storytelling, embodiment, and shared experience, Playback Theatre has been applied across diverse contexts, including therapy, education, healthcare, and social work. Studies have demonstrated its efficacy in trauma recovery, conflict resolution, and personal empowerment, as it provides a structured yet flexible space for individuals to explore difficult emotions in a supportive environment [12]. Additionally, its role in training professionals, such as therapists, educators, and healthcare workers, has been recognized for enhancing empathy, communication skills, and emotional intelligence [13]. Despite its growing application across therapeutic and educational contexts, empirical understanding of individual lived experiences in Playback Theatre remains limited. Previous research has mainly focused on its potential psychological and social effects in general terms, but few studies have explored how participants actually experience, interpret, and embody these effects in practice. By examining participants’ reflections through a phenomenological approach, this study aims to illuminate the experiential processes that give rise to Playback Theatre’s psychological and social significance. We also emphasize that the three theoretical frameworks discussed earlier (self-reflection, embodied cognition, and social mirroring) provide partial explanations of its mechanisms but do not capture the subjective, first-person meaning-making that underlies them. Hence, a phenomenological approach was chosen to explore these lived experiences and to bridge these theoretical domains through participants’ own voices.

This study explores participants’ lived experiences of Playback Theatre, focusing on how they perceive and make sense of the process in relation to self-awareness, empathy, and social connection. Specifically, it examines (1) how participants experience self-awareness and personal insight, (2) how they experience understanding and empathy toward others, and (3) how they reflect on the overall psychological and social significance of Playback Theatre. While the structure of Playback Theatre provides the setting for these experiences, the present study primarily addresses participants’ internal meaning-making processes rather than the performative mechanisms themselves. By addressing these aspects, this study contributes to the growing body of research on expressive arts therapies, embodiment in emotional processing, and the psychology of storytelling. Moreover, it offers valuable insights for practitioners who use Playback Theatre in therapeutic, educational, and professional development settings. Understanding how individuals experience and interpret Playback Theatre can help refine its application as a tool for psychological exploration and social connection.

The significance of this research lies in its ability to bridge the fields of psychology, performance studies, and social interaction. While Playback Theatre has been widely practiced, empirical research on its impact from a first-person, experiential perspective remains limited. By employing a phenomenological approach, this study provides a detailed exploration of participants’ lived experiences, offering a nuanced understanding of how Playback Theatre fosters personal transformation and communal empathy.

## Materials and methods

This study is a qualitative phenomenological study employing Interpretive Phenomenological Analysis (IPA) to examine how participants experience and make sense of their engagement in Playback Theatre. IPA is a qualitative research approach that aims to explore individuals’ lived experiences, emphasizing their subjective interpretations and personal meaning-making processes [14,15]. Given the introspective and transformative nature of Playback Theatre, IPA is well-suited to capture the depth of emotional, cognitive, and embodied experiences reported by participants.

Overview of Playback Theatre

Playback Theatre is a participatory form of improvisational theatre that transforms personal stories into spontaneous performances. Developed by Jonathan Fox and Jo Salas in the 1970s, it combines elements of storytelling, embodiment, and collective reflection [1-4]. In Playback Theatre, a participant, called the teller, shares a personal story in front of other participants. The conductor (facilitator) guides the teller through a brief dialogue to clarify the narrative, after which a group of actors immediately reenact the story using movement, gesture, and spontaneous dialogue, without any prior rehearsal. The themes of these narratives vary widely, ranging from everyday encounters to experiences of conflict, suffering, or personal transformation. Through this process, Playback Theatre transforms individual experiences into shared, embodied performances, allowing both tellers and witnesses to reflect on the emotional and relational meanings of their stories [1-4]. Fox states that the purpose of Playback Theatre is to educate through the stories people share and the connections they create [1]. He emphasizes that this knowledge differs from technical, scientific, or professional expertise. Whereas technical or scientific knowledge is characterized by analytical reasoning and objective procedures, the knowledge cultivated through Playback Theatre is experiential, relational, and embodied. It emerges through participants’ lived engagement, listening to others’ stories, witnessing their enactment, and reflecting together on the meanings that surface in those moments. This form of knowledge involves empathic understanding and mutual attunement, rather than formal instruction, and thus resonates with phenomenological approaches that value direct, first-person experience as a source of insight. Furthermore, he notes that everyday professional life often lacks opportunities for interaction through personal storytelling, limiting access to this form of knowledge.

Participants

A total of 44 distinct individuals participated in this qualitative phenomenological study across six Playback Theatre sessions held in Tokyo between January and August 2018. While a few participants attended more than one session, each individual was counted only once in the total number and demographic summaries to avoid duplication. Participants were recruited through purposive and voluntary sampling, as the study aimed to capture diverse perspectives on personal experiences with Playback Theatre. Inclusion criteria were as follows: (1) adults aged 18 years or older, (2) attendance at one of the Playback Theatre sessions organized by the research team, (3) willingness to provide written reflections about their experiences, and (4) ability to communicate in Japanese. Exclusion criteria included individuals experiencing acute psychological distress or those who did not provide informed consent. Details of participant demographics are presented in the Results section.

Study design and procedure

The Playback Theatre sessions in this study were planned and implemented as naturalistic workshops collaboratively organized by the research team and a certified Playback Theatre ensemble. Each session was facilitated by certified Playback Theatre practitioners, including one conductor and three to five actors, all of whom had completed formal Playback Theatre training and possessed at least five years of performance experience. The practitioners were members of a recognized Playback Theatre ensemble and were purposefully invited by the research team to ensure that the sessions were conducted according to established Playback Theatre principles and with appropriate attention to psychological safety. The actors, in contrast, were not professional performers but participants in each workshop, who took turns enacting the tellers’ stories spontaneously. In each session, three to five participants served as tellers, sharing personal stories that were then enacted by other participants. This format reflected the participatory essence of Playback Theatre, in which storytelling and performance are collectively created by the participants themselves under the conductor’s guidance.

The sessions were conducted in Tokyo between January and August 2018. Participants were recruited through open calls and personal invitations distributed among community groups, educators, and healthcare professionals who had expressed interest in expressive arts workshops. Participation was entirely voluntary. Each session lasted approximately two to two and a half hours, consisting of warm-up exercises (about 30 minutes), storytelling and enactment (about 60-75 minutes), and reflective dialogue (about 30-45 minutes). The authors designed the study protocol and attended the sessions primarily as observers and coordinators, ensuring smooth operation and data collection, but they did not serve as performers or conductors to minimize potential bias. No pre-defined themes were provided in advance; participants were invited to share any personally meaningful experience they wished, which was then enacted by the practitioners in real time according to standard Playback Theatre practice.

The authors designed and coordinated the overall study but did not serve as conductors or actors during the Playback Theatre sessions. All sessions were facilitated by certified Playback Theatre practitioners who were independent of the research team. The authors attended the sessions as observers and coordinators, ensuring smooth operation, ethical oversight, and data collection while minimizing potential bias or influence on participants’ experiences.

After each session, participants were invited to provide short written reflections in response to three open-ended questions concerning: (1) self-awareness and personal transformation, (2) reflections on others and interpersonal resonance, and (3) overall impressions of the Playback Theatre experience. In this study, the term “response” refers to one written reflection submitted by a single participant for one of these three questions. Each participant could therefore contribute up to three responses, depending on whether they addressed all questions. A total of 127 responses were collected from 44 participants across six sessions. Although most participants provided reflections on all three questions, a few did not complete every item, resulting in slight differences in the number of responses by question (43, 41, and 43, respectively). These reflections were treated as individual meaning units in the Interpretative Phenomenological Analysis (IPA). No in-depth interviews were conducted, and the enacted performances themselves were not treated as analytic data.

Data analysis

The collected data were analyzed following the standard IPA framework [15]: (1) reading and re-reading, in which each response was read multiple times to ensure deep engagement with the data; (2) initial coding and identifying emergent themes, wherein key phrases and concepts that reflected participants’ subjective experiences were highlighted; (3) clustering themes into superordinate categories, in which recurring patterns across individual accounts were grouped into broader thematic categories; and (4) interpretation and integration, in which the emergent themes were contextualized with existing theories of self-reflection, embodied cognition, and social mirroring to provide a deeper understanding of the participants’ experiences.

The concept of self-reflection was informed by Schön’s notion of reflective practice [16] and Mezirow’s theory of transformative learning [17], which emphasize critical awareness arising from personal experience. The perspective of embodied cognition drew on Gallese and Lakoff [8], highlighting how cognitive and emotional understanding are grounded in bodily experience. Finally, social mirroring was interpreted in light of Gallese’s theory of embodied simulation [10] and Meltzoff and Decety’s work on shared neural representations underlying empathy and intersubjectivity [18]. These theoretical lenses provided interpretive depth for understanding how participants experienced self-awareness, embodied empathy, and social attunement through Playback Theatre.

In this study, Playback Theatre was conceptualized as both a phenomenon of individual experience and a contextual framework that shaped those experiences. The analysis, therefore, attended to two interrelated dimensions: (1) participants’ lived experiences of engaging in Playback Theatre and (2) the ways in which the structure and process of Playback Theatre itself facilitated those experiences. Interpretative Phenomenological Analysis (IPA) was particularly suited to examining this relationship, as it allows researchers to explore how individuals interpret their experiences within a given social and embodied context. Through iterative interpretation and dialogue among the researchers, the analysis sought to illuminate not only what participants experienced but also how the conditions of Playback Theatre enabled those experiences to emerge.

Both authors independently read all written reflections multiple times to gain familiarity with the data and identify preliminary meaning units. Each author then conducted open coding and developed emergent themes individually. The two sets of codes were subsequently compared and discussed in detail until consensus was reached, ensuring investigator triangulation. The analysis was further refined through theoretical triangulation, in which the emerging themes were interpreted in relation to three theoretical perspectives: self-reflection, embodied cognition, and social mirroring. Throughout the process, the first author (a clinician-educator/qualitative researcher with expertise in phenomenological methods) contributed interpretive insights grounded in clinical and educational practice, while the second author (a clinician familiar with Playback Theatre) provided methodological oversight and reflexive evaluation. These iterative dialogues among the researchers enhanced the credibility and depth of the findings. The final themes were determined based on their prevalence across responses and their alignment with the study’s research questions.

The core themes were derived inductively from participants’ written reflections using Interpretative Phenomenological Analysis (IPA). Theoretical perspectives, including self-reflection, embodied cognition, and social mirroring, were not imposed a priori but were used later to interpret and contextualize the findings, providing theoretical depth to the experiential meanings identified in the data.

The first author (DS), a clinician and medical educator with expertise in phenomenological qualitative analysis, brought an applied and empathic perspective to the interpretation of participants’ experiences. The second author (MS), a qualitative researcher with prior experience in expressive arts and Playback Theatre, contributed methodological rigor and reflexive oversight to the analytic process. Through ongoing dialogue between these complementary perspectives, the analytic process became both critically self-aware and interpretively balanced, reinforcing the study’s credibility and depth.

Ethical considerations

Participation in the study was voluntary, and informed consent was obtained from all participants. They were assured that their reflections would remain anonymous, and all identifying information was removed from the dataset. The study was conducted in accordance with ethical guidelines for research involving human participants, ensuring that no psychological harm resulted from participation. The study was conducted in accordance with the Declaration of Helsinki [19] and adhered to ethical guidelines for research involving human participants. This study was approved by the Ethics Review Committee of the University of Tokyo School of Medicine (approval number: 10994).

## Results

Participant characteristics

Table 1 presents a summary of the sessions, including the number of participants, mean age, and age range. The number of participants per session varied from 4 to 10 individuals, with an overall mean age of 41 years (ranging from 17 to 60 years). Participants came from diverse backgrounds, including healthcare professionals, educators, therapists, artists, and individuals with no prior experience in theatre or psychotherapy. This heterogeneity provided a broad perspective on how different individuals engage with and respond to Playback Theatre.

**Table 1:** Summary of Playback Theatre Sessions and Participant Demographics SD: standard deviation

Session	Date	Place	Number of Participants (Percent)	Age (Mean (SD))	Age (Range)
1	January, 2018	Tokyo	10 (22.7)	38.2 (10.4)	17-50
2	March, 2018	Tokyo	9 (20.5)	40.5 (10.2)	27-58
3	April, 2018	Tokyo	9 (20.5)	41.2 (13.1)	21-60
4	May, 2018	Tokyo	7 (15.9)	40.7 (9.6)	31-58
5	June, 2018	Tokyo	4 (9.1)	39.5 (1.9)	38-42
6	August, 2018	Tokyo	5 (11.4)	47.6 (8.9)	38-60
Total	-	-	44 (100)	41.0 (10.1)	17-60

A total of 127 written reflections were collected from 44 participants who attended six Playback Theatre sessions. Participants provided reflections in response to three open-ended questions: (1) self-awareness and personal transformation, (2) reflections on others and interpersonal resonance, and (3) overall impressions of the Playback Theatre experience. While most participants responded to all three questions, a few did not complete every item, resulting in slight variation in the number of responses across the categories (43, 41, and 43, respectively). These reflections were analyzed as individual meaning units through Interpretative Phenomenological Analysis (IPA).

Interpretive Phenomenological Analysis (IPA) of participants’ experiences in Playback Theatre

We conducted an Interpretive Phenomenological Analysis (IPA) to examine how individuals made sense of their experiences and to identify core themes related to self-awareness, interpersonal connections, and emotional transformation. The participants’ reflective writings were structured according to three guiding research questions, which also provided the framework for subsequent analysis. Each research question corresponded to one major analytical dimension identified through Interpretative Phenomenological Analysis (IPA). Specifically, the first question regarding self-awareness and personal insight was associated with the dimension self-awareness and personal transformation, the second question concerning understanding and empathy toward others aligned with reflections on others and interpersonal resonance, and the third question addressing the overall psychological and social impact of Playback Theatre corresponded to overall reflections on the Playback Theatre experience (Table 2). This correspondence ensured conceptual coherence between the study’s design and its emergent findings.

**Table 2:** Themes and Participants’ Perspectives in Playback Theatre

Dimensions	Themes	Participants’ Perspectives (Quotes)
A. Self-Awareness and Personal Transformation	Recognizing and Moving Beyond Personal Boundaries	- “I realized how passive I usually am.” - “Opening up about myself was challenging.” - “I discovered that crying in front of others is not necessarily a bad thing.”
	Gaining New Insights Into Oneself	- “I found that I actually have more energy inside me than I thought.” - “This experience allowed me to reflect on my emotions and thoughts in ways I hadn’t before.”
	Transformation Through Embodied Experience	- “At first, I was shy about expressing myself physically, but I eventually felt liberated.” - “Removing language opened up a new world of expression for me.”
	Experiencing Safety and Trust in Encouraging Exploration	- “I felt at ease here, which helped reduce my usual anxiety.” - “This was a rare chance to express myself without hesitation.”
B. Reflections on Others and Interpersonal Resonance	Admiration for Others’ Expressiveness and Presence	- “I was overwhelmed by the richness of expression, improvisation, and flow.” - “Seeing someone fully immerse themselves in a role was inspiring.”
	Recognizing the Vulnerability and Strength in Others	- “I caught a glimpse of her true feelings during a shouting exercise.” - “I sensed that some people were holding back a part of themselves, just like I do.”
	Experiencing Mutual Connection and Unexpected Resonance	- “Somehow, I kept ending up in the same group with certain people, and it felt significant.” - “Doing an expressive exercise with a near-stranger made me feel unexpectedly close to them.”
	The Impact of Playback Theatre in Deepening Understanding of Others	- “By playing the role of a ‘villain,’ I realized that even those who hurt us have their own struggles.” - “Playback Theatre blurred the lines of age, profession, and background; we were just people experiencing together.”
C. Overall Reflections on the Playback Theatre Experience	The Power of Being Seen, Heard, and Acknowledged	- “Seeing someone’s past being reenacted so tenderly was moving.” - “Playback Theatre gives weight to the moments we have lived, making them feel irreplaceable.”
	The Role of Structure and Safety in Facilitating Expression	- “The way barriers were gradually removed throughout the session was fascinating.” - “I now understand what it means for a space to cultivate sharing and openness.”
	The Physical and Emotional Release Through Movement	- “It was refreshing to move rather than just think; my body felt recharged.” - “The interplay of movement, silence, and performance created an almost meditative space.”
	The Uniqueness and Ephemerality of Playback Theatre	- “Each session is completely unique, depending on the people, place, and timing.” - “I was struck by how different this felt compared to my previous experiences.”

The following section explains the core themes identified through IPA, categorized into three dimensions. These themes were subsequently aligned with the corresponding research questions to provide a structured account of the participants’ lived experiences.

Self-Awareness and Personal Transformation

Playback Theatre served as a catalyst for self-discovery and transformation, allowing participants to gain new insights into themselves, their limitations, and their potential. The thematic analysis revealed four key areas.

Recognizing and moving beyond personal boundaries: Participants described becoming aware of personal boundaries, such as hesitation, self-doubt, or emotional restraint, that had often gone unnoticed in daily life. Through the process of storytelling and enactment, several participants reflected that they came to see these limits differently, sometimes feeling a gradual loosening or transformation rather than a dramatic “breakthrough.” This theme reflects participants’ own meaning-making about growth and emotional openness fostered by the shared performance experience.

Gaining new insights into oneself: Participants frequently noted an enhanced understanding of their emotions, energy levels, and self-perception. Through engagement in expressive activities, they reflected on internalized patterns and discovered aspects of themselves they had not previously acknowledged.

Transformation through embodied experience: The non-verbal, physical aspects of Playback Theatre played a significant role in participants’ transformation. Movement and physical expression were described as unlocking emotional release and fostering deeper self-awareness. Participants noted that their connection with others often occurred non-verbally, through eye contact, mirroring, and shared movement, which conveyed emotions more powerfully than words. These subtle interactions created a sense of attunement and embodied empathy, deepening mutual understanding within the group.

Experiencing safety and trust in encouraging exploration: Rather than perceiving the space as externally “safe,” participants described feeling accepted and trusted within the group. This sense of safety emerged through mutual listening and shared vulnerability, which allowed participants to express emotions more freely. The experience of relational safety was described as integral to their ability to reflect and connect with others.

Reflections on Others and Interpersonal Resonance

A significant aspect of Playback Theatre involved participants’ interactions with and observations of others. These reflections provided insights into how participants experienced empathy, admiration, and connection in a shared space.

Admiration for others’ expressiveness and presence: Many participants expressed admiration for the artistic and expressive abilities of others. Witnessing performances allowed them to appreciate different styles of emotional expression.

Recognizing the vulnerability and strength in others: Playback Theatre facilitated a deeper awareness of the complexity of human emotion. Participants observed moments of vulnerability and strength in others, which, in turn, made them more aware of their own internal experiences.

Experiencing mutual connection and unexpected resonance: Participants described moments where they unexpectedly resonated with others, whether through shared experiences or spontaneous emotional alignment. Many noted that gestures, tone, and movement created a sense of moving together or “feeling the same rhythm,” which fostered a shared emotional atmosphere. These experiences represent a form of social attunement, an embodied synchronization with others that emerged through the collective process of storytelling and enactment.

The impact of Playback Theatre in deepening understanding of others: Participants described how their understanding of others deepened through embodied and emotional engagement in Playback Theatre. Rather than a sudden realization, this was experienced as a felt connection while witnessing or re-enacting others’ stories. As they embodied others’ emotions and listened to vulnerable narratives, participants became aware of shared feelings and recognized a sense of common humanity, a lived, empathic deepening rather than a cognitive conclusion.

Overall Reflections on the Playback Theatre Experience

Participants’ overall impressions of Playback Theatre revealed an appreciation for the unique combination of storytelling, embodiment, and communal engagement. The following themes emerged.

The power of being seen, heard, and acknowledged: Playback Theatre provided a space where participants felt deeply validated. The act of having their stories reenacted, or simply witnessing others’ stories, created a sense of emotional recognition and appreciation.

The role of structure and safety in facilitating expression: Participants viewed the structured flow and sense of safety in Playback Theatre as key to enabling self-expression. The predictable sequence and guidance of a trusted conductor created stability and mutual trust, easing anxiety about sharing personal stories. Safety was experienced as emerging through attentive listening and mutual responsiveness, allowing participants to express previously suppressed emotions and feel relief and connection within this supportive framework.

The physical and emotional release through movement: The embodied nature of Playback Theatre created an intuitive and cathartic space that facilitated emotional processing through movement rather than words.

The uniqueness and ephemerality of Playback Theatre: Several participants reflected on how each session was deeply unique and unrepeatable due to the spontaneous nature of storytelling and performance.

The analysis highlights several key impacts. Participants encountered aspects of themselves they had not previously recognized, often experiencing a shift in how they viewed their own emotions and limitations. Through storytelling, participants realized how much their experiences resonated with others, fostering a deep sense of mutual understanding and recognition. The use of movement and physical expression provided a new way of processing emotions, with many participants finding non-verbal communication to be just as meaningful as words. Additionally, the secure environment created by Playback Theatre encouraged participants to take emotional risks, allowing them to feel free, unburdened, and fully present in the experience.

Playback Theatre is not just an improvisational performance but a transformative, reflective, and deeply human experience. It provides a rare opportunity to break free from daily constraints, facilitating both self-acceptance and collective empathy. Through storytelling and embodied engagement, participants experience an immersive form of introspection and connection, leading to profound shifts in self-awareness and interpersonal understanding.

## Discussion

In this discussion, we first interpret the participants’ lived experiences of Playback Theatre as revealed through Interpretative Phenomenological Analysis (IPA). We then examine the significance of these findings by relating them to existing theories of self-reflection, performative therapy, and social connectedness, highlighting how Playback Theatre facilitates meaning-making and empathy through embodied and dialogic engagement. By situating our results within prior research, we aim to clarify the unique contribution of this study to understanding the psychological and social dimensions of participatory theatre practices.

The findings from the Interpretive Phenomenological Analysis (IPA) of Playback Theatre participants reveal its profound role as a tool for self-discovery, emotional expression, and interpersonal connection. The experience was not merely an artistic or recreational activity but a deeply transformative engagement that was facilitated by psychological safety, empathy, and embodied self-awareness. In this discussion, we examine the significance of these findings in relation to existing theories of self-reflection, performative therapy, and social connectedness, as well as implications for future research and practice.

Playback Theatre as a catalyst for self-discovery and emotional processing

One of the most striking aspects of the findings was the way Playback Theatre facilitated self-discovery and transformation. Many participants described recognizing and moving beyond personal boundaries, uncovering hidden aspects of themselves, and gaining a newfound sense of agency over their emotional expression.

Theatre, especially in its improvisational forms, has long been recognized as a powerful tool for self-reflection and self-concept development [12]. Playback Theatre is unique in that it requires participants to witness their own narratives being played back, leading to a form of mirroring and externalization. This process aligns with theories of psychodrama and expressive therapy, which posit that performing personal experiences allows individuals to gain cognitive and emotional distance, facilitating deeper reflection [20].

The findings support these theoretical underpinnings, as participants frequently reported moments of realization about their emotional tendencies, fears, and strengths. The act of seeing their experiences performed gave weight to their personal narratives, reinforcing a sense of validity and self-acceptance.

A critical factor in enabling this transformation was the presence of a context-specific sense of safety experienced by participants. While this may resemble the concept of psychological safety as proposed in organizational contexts [21], the form of safety described here was relational and embodied, grounded in mutual trust, attentive listening, and non-judgmental acceptance within the group. Participants described this sense of safety as felt through interaction, a condition that allowed emotional openness and expressive freedom in the Playback Theatre process. Participants reported that the structured, supportive, and non-judgmental environment encouraged them to take emotional risks. This aligns with research on safe spaces in expressive therapies, which highlights that individuals are more likely to engage in deep self-exploration when they feel protected from judgment and external pressures [22].

Interestingly, the findings also indicated that some participants initially felt hesitant or fearful of revisiting their past experiences. This suggests that while Playback Theatre is a powerful tool for self-reflection, it must be facilitated with care and sensitivity, ensuring that participants are emotionally prepared for the depth of engagement it entails.

Interpersonal connection and empathy development

Another key finding was the deep sense of connection participants felt with others. Through storytelling and enactment, Playback Theatre created a space for mutual recognition, emotional resonance, and shared vulnerability.

Participants frequently described moments of unexpected connection, where another person’s story felt intimately familiar or resonated deeply with their own experiences. This supports theories of social mirroring and collective empathy, which suggest that witnessing another’s experience being performed can trigger a process of internalization and identification [10]. Merleau-Ponty provides a phenomenological explanation for this phenomenon in his discussion of intercorporeality (inter-bodily relation), where he argues that our experience of the world is always intertwined with that of others [23]. He further elaborates that perception is not solely an individual cognitive act but emerges through embodied interaction [23]. Playback Theatre thus serves as a medium for social attunement, allowing participants to realize that their personal struggles and emotions are not isolated but part of a broader human experience.

This process is particularly relevant in the context of therapeutic group work. Studies on group psychotherapy highlight that shared emotional experiences can enhance belonging and reduce emotional isolation [13]. In our findings, this phenomenon, the emergence of emotional connectedness and mutual understanding among participants, appeared to arise within the structured yet open framework of Playback Theatre. Although the sessions were intentionally facilitated through a defined sequence of storytelling and enactment, the sense of belonging and resonance developed organically through participants’ shared engagement and embodied empathy. In this way, Playback Theatre provides an intentionally supported but experientially emergent form of community-building and psychosocial support.

Another significant theme was the embodied nature of empathy experienced by participants in Playback Theatre. Several participants described moments of bodily resonance and interconnectedness, feeling “moved together” with others or sensing emotions through physical gestures and shared rhythm. For actors, empathy was experienced through embodied enactment, as they physically and emotionally inhabited another person’s story, often noticing subtle shifts in posture, breath, or tone that evoked shared emotion. For tellers and witnesses, empathy was likewise felt in the body, for example, through tears, chills, or an urge to move, while watching their own or others’ stories being enacted. These accounts suggest that Playback Theatre fosters a multidimensional embodied empathy, in which emotional understanding arises through bodily engagement, mutual resonance, and relational presence.

Research on embodied cognition suggests that physical enactment enhances emotional understanding by allowing individuals to simulate and internalize another’s experience at a physiological level [8]. This finding aligns with participants' descriptions of feeling as though they momentarily “became” the person whose story they were enacting. It suggests that Playback Theatre could be an effective intervention for enhancing empathy in professions that require high emotional intelligence, such as healthcare, counseling, and education.

The role of movement and non-verbal communication

Playback Theatre is distinguished by its emphasis on movement, physicality, and non-verbal expression. The findings indicate that for many participants, physical enactment provided a means of emotional release and self-understanding that words alone could not achieve.

Many participants expressed surprise at how movement unlocked emotions they had not consciously processed. This aligns with somatic theories of trauma processing and expressive therapy, which propose that certain emotions are stored in the body and may not be easily accessed through verbal reflection alone [9]. Playback Theatre’s integration of bodily expression thus provides an alternative, sensorimotor pathway for emotional expression, relief, and integration.

Additionally, participants highlighted that non-verbal elements, such as eye contact, mirroring, and spatial relationships, often conveyed more meaning than words. This is consistent with studies on non-verbal communication, which suggest that up to 93% of emotional meaning is conveyed through non-verbal cues [24]. These findings underscore the importance of non-verbal storytelling as a means of exploring complex emotions.

The uniqueness and potential applications of Playback Theatre

A final key finding was the recognition of the individual and diverse nature of participants’ experiences within Playback Theatre. Participants frequently commented on how the experience felt deeply personal yet universally resonant. Although sessions followed a common structure, participants’ reflections revealed distinct emotional tones and perspectives, each story and response carrying its own meaning and resonance. This individuality highlights how Playback Theatre enables personally unique experiences of expression and connection, even within a shared framework.

The transient nature of Playback Theatre, where each performance is improvised and unrepeatable, contributed to a heightened sense of presence and engagement. This is consistent with theories of performative consciousness, which argue that ephemeral experiences hold a heightened sense of authenticity and emotional significance [25].

Given its powerful effects on self-awareness, empathy, and emotional expression, Playback Theatre has significant potential applications beyond its current artistic setting. Prior work suggests that enactment can externalize and organize overwhelming affect in trauma-related contexts [5,9] and that expressive arts modalities support integration and regulation [7]. Our findings add a phenomenological account of how participants experience this process as a gradual loosening of emotional boundaries and relational support, indicating a low-threshold, community-based complement to formal psychotherapy. In medical and healthcare training, arts-based and theatre-in-education approaches have been associated with enhanced empathy and perspective-taking [26,27]. Our data specify how empathy is felt as embodied resonance (eye contact, mirroring, and shared rhythm), suggesting Playback Theatre as a practical exercise for sensorimotor perspective-taking, alongside standardized patient work. In education and social work, applied/participatory theatre has been used to explore identity, diversity, and social dynamics [28,29]. Building on this, our results indicate that students/community members can co-create psychological and relational safety and articulate personal meanings through shared enactment, pointing to applications in classroom climate building and community psychosocial support.

Strengths and limitations

This study has several methodological strengths. The use of Interpretative Phenomenological Analysis (IPA) provided a systematic yet flexible framework for exploring participants’ lived experiences in depth. Collecting written reflections immediately after each session allowed the data to capture spontaneous and authentic emotional responses, minimizing retrospective bias. In addition, investigator triangulation and reflexive dialogue between the authors enhanced the credibility and interpretive rigor of the analysis. At the same time, several limitations should be noted, such as the single-site design, the reliance on self-reported reflections, and the limited generalizability inherent in qualitative research.

However, several limitations should also be noted. First, the study involved 44 participants from sessions conducted in Tokyo. As this research employed a qualitative, experience-centered design, its findings are not intended for statistical generalization, but rather for a nuanced understanding of individual experiences within a specific cultural context. This design inherently limits what can be shown in breadth, while allowing for depth and richness in the interpretation of lived experiences. Second, because participation was voluntary and data were collected through subjective reflections, there is a possibility of self-selection and reporting bias, where participants may have emphasized positive experiences while minimizing challenges. Lastly, the study captured only immediate reflections following the sessions, leaving the longer-term psychological and social effects of Playback Theatre unexplored. Future research should address these limitations by including more diverse participant groups, incorporating objective measures, and conducting longitudinal studies to assess the sustained impact of Playback Theatre over time.

## Conclusions

This study highlights Playback Theatre as a powerful tool for self-awareness, emotional expression, and interpersonal connection. The findings suggest that the experience fosters psychological safety, deep reflection, and embodied empathy, allowing participants to engage in self-discovery while resonating with others’ stories. Through its unique blend of movement, storytelling, and improvisation, Playback Theatre enables participants to break through personal limitations, process emotions, and develop a profound sense of shared humanity. Ultimately, Playback Theatre is more than performance: it is a transformative, immersive experience that bridges personal growth with collective empathy and human connection.
